# A Cost-Effective CNN-LSTM-Based Solution for Predicting Faulty Remote Water Meter Reading Devices in AMI Systems

**DOI:** 10.3390/s21186229

**Published:** 2021-09-17

**Authors:** Jaeseung Lee, Woojin Choi, Jibum Kim

**Affiliations:** Department of Computer Science and Engineering, Incheon National University, Incheon 22012, Korea; hunni10@inu.ac.kr (J.L.); 202027009@inu.ac.kr (W.C.)

**Keywords:** machine learning, advanced meter infrastructure (AMI), CNN-LSTM, deep learning, water, fault detection

## Abstract

Automatic meter infrastructure (AMI) systems using remote metering are being widely used to utilize water resources efficiently and minimize non-revenue water. We propose a convolutional neural network-long short-term memory network (CNN-LSTM)-based solution that can predict faulty remote water meter reading (RWMR) devices by analyzing approximately 2,850,000 AMI data collected from 2762 customers over 360 days in a small-sized city in South Korea. The AMI data used in this study is a challenging, highly unbalanced real-world dataset with limited features. First, we perform extensive preprocessing steps and extract meaningful features for handling this challenging dataset with limited features. Next, we select important features that have a higher influence on the classifier using a recursive feature elimination method. Finally, we apply the CNN-LSTM model for predicting faulty RWMR devices. We also propose an efficient training method for ML models to learn the unbalanced real-world AMI dataset. A cost-effective threshold for evaluating the performance of ML models is proposed by considering the mispredictions of ML models as well as the cost. Our experimental results show that an F-measure of 0.82 and MCC of 0.83 are obtained when the CNN-LSTM model is used for prediction.

## 1. Introduction

Water is one of the most important resources for mankind. However, global desertification and water pollution have led to a steep rise in water prices. A stable water supply has become a very important concern for the general population as well as water suppliers. Most water suppliers are governments or municipalities, and non-revenue water (NRW) is an important concern for those water suppliers. NRW refers to water that has been supplied but not billed. Some of the main causes of NRW are faulty water meters, water leakage, and theft. Minimizing NRW is important, as it is directly related to the revenues earned by water suppliers. The NRW varies from region to region; it is less than 5% in countries such as Singapore and more than 70% in some parts of Africa [1].

AMI systems use remote water metering to record all water consumption during short time intervals. These recorded values are transmitted to a server through a wireless network and stored for each customer. There are several advantages in using the AMI remote water meter system compared to the existing water meter system. First, it efficiently manages water resources and reduces NRW through real-time monitoring by locating failures/faults in the remote water meter reading (RWMR) devices before a meter-reading employee physically visits and checks them. Faulty RWMR devices are one of the main causes of NRW because the water consumption recorded may be less than the amount of water actually used owing to a failure/fault. Sometimes, even when water has been used, zero consumption may be recorded. Bursting of water pipes, another cause of NRW, can also be detected using real-time monitoring of AMI system [2].

Second, it is possible to reduce costs using the AMI system because meter-reading employees do not have to visit the meter in person in most cases. In particular, if the number of customers increases substantially in the future, water suppliers may face a shortage of manpower and the labor cost of the meter-reading staff may increase considerably. The AMI system using remote metering solves this problem. Finally, water suppliers can provide various additional services to customers by utilizing big data on water consumption collected from their customers. It is possible to provide a user-customized rate plan as well as services such as an alarm for the elderly living alone and an alarm for freezing.

**Figure 1:**
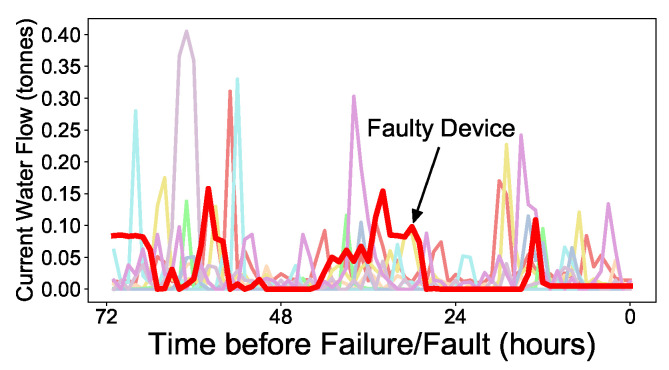
Example of current water flow (consumption) of faulty (bold red) and healthy RWMR devices (other colors) prior to failure/fault.

If a faulty RWMR device can be accurately and quickly predicted, the problematic device can be checked and replaced before the actual failure/fault occurs, thereby reducing the expected NRW in the future. For water suppliers such as local governments, complaints from customers that may arise from problematic devices can also be substantially reduced. Therefore, there is a need for a complete solution that can accurately identify faulty RWMR devices. However, our AMI data only stores limited information such as water flow (consumption) because it uses the AMI battery and faces other hardware issues. Therefore, it is not easy to distinguish between a healthy device and a faulty device based on the limited information collected. Figure 1 shows a small example. We observe that the current water flow (consumption) pattern of a faulty device may not differ significantly from that of a healthy device. To overcome this issue, we propose a complete CNN-LSTM-based solution that can accurately predict the faulty RWMR device. In particular, we propose a method to evaluate the performance of ML models by considering the costs arising from the misprediction of the ML models.

There have been many studies on hard disk drive (HDD) failure prediction for machine failure, and many failure prediction models have been developed [3,4]. If the HDD fails, data loss may occur and users may face economic loss. To prevent this, most HDD manufactures have implemented self-monitoring, analysis, and reporting technology (SMART). SMART determines an HDD’s health by collecting various hard disk parameters such as power-on hours and temperature [4]. Although this SMART software provides a basic evaluation, it simply indicates that the HDD is likely to fail soon if any parameter exceeds the threshold. In addition, the threshold value of each of these parameters is confidential because only the manufacturer knows it, and the manufacturer has to replace the HDD in case of failure.

The AMI data used in this study has several differences from the SMART dataset, which is often used for HDD failure prediction. First, the SMART dataset is an indicator used by manufacturers to predict HDD failure, and there are many meaningful features that can be used for ML models. As described above, these meaningful features are set by the HDD manufacturer, and based on threshold, if any feature exceeds the threshold value, the model warns that the HDD will fail soon. The SMART dataset, which is widely used in research, has 60 features [3]. By contrast, the AMI data collected and stored in this study only includes limited features such as pipe diameter, water flow (consumption), and AMI battery voltage value. This is because, unlike the HDD dataset, water meter AMI data uses a battery, so battery consumption must be considered, and therefore only limited information is collected and stored.

Only a few studies predict the failure/fault of AMI remote water meters or remote water meter reading devices in an actual AMI system. One study attempted to find leaks and theft in the common areas and faulty meters through machine learning by analyzing AMI data in apartment buildings [1]. Another study attempted fault detection using a data-driven method using kernel principal component analysis (PCA) [5]. In another work [6], the authors analyze the AMI data using machine learning in the apartment, focusing on detecting leaks.

Unlike previous studies, we analyze big data, approximately 2,850,000 AMI data, collected from 2,762 customers for 360 days in a small-sized city in South Korea and predict the failure/fault of the RWMR device using ML models. The main contribution of our paper is summarized as follows:

- Real AMI data is collected and stored using RWMR devices in a small-sized city in South Korea. A complete CNN-LSTM-based solution that can predict failures/faults in RWMR devices is proposed. A CNN-LSTM model is first used for predicting faulty RWMR devices, and the experimental results show high prediction results.
- The AMI data used in this study has many missing values and limited features. Furthermore, it is a highly unbalanced dataset where the number of healthy data is much higher than the number of faulty data. We introduce an extensive preprocessing method and propose solutions for ML models to learn this challenging real-world dataset.
- We perform extensive experiments to demonstrate that the CNN-LSTM model is the most appropriate for predicting faulty RWMR devices in terms of prediction accuracy and efficiency. We also propose a cost-effective threshold for evaluating the performance of ML models.

The rest of the article is organized as follows. In Section 2, detailed descriptions of the AMI data used in this study are introduced. In Section 3, extensive preprocessing methods are described for the AMI data. In Section 4, we introduce the ML models used in the experiment. In Section 5, the dataset and metrics used in the experiment are explained. Section 6 shows experimental results. We conclude our paper in Section 7.

## 2. Automatic Meter Infrastructure (AMI) Data

### 2.1. AMI Data Collection and Storage Process

**Figure 2:**
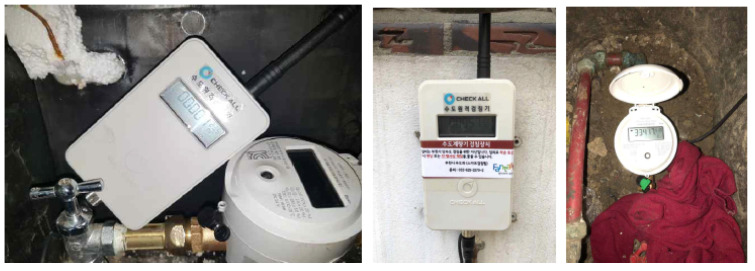
The left is the RWMR device installed in the household. The center is the AMI remote meter reader and the right is the water meter for remote meter reading.

In this study, AMI data are collected from RWMR devices installed in a small-sized city in South Korea. Here, the RWMR device is composed of a water meter used for remote meter reading and an AMI remote meter reader. Figure 2 shows a RWMR device installed in a household. Using the water meter for remote meter reading, AMI data are collected from each customer for every hour (e.g., 12:00). The collected AMI data is stored in the server through the base station of the telecommunication company and the Internet-of-Things (IoT) platform through the Narrowband (NB)–IoT communication network using the AMI remote meter reader. All AMI data are collected by an IS technology system (More information regarding IS technology’s Check All system can be found at http://istec.co.kr/(accessed on 10 September 2021)).

### 2.2. AMI Data Description

The following values are stored in the AMI data: (1) customer ID (2) measurement time (3) status code (4) pipe size (5) AMI battery voltage (6) cumulative water flow in tonnes (7) current water flow in tonnes. Here, the water flow corresponds to the customer’s water consumption.

Each water meter basically captures measurements at 1:00, 2:00, 3:00, 4:00, and so on, and the status of the RWMR device is stored in the AMI data status code. The status of the RWMR device is basically classified as healthy (label 0) or faulty (label 1). Here, healthy means that the RWMR device is operating normally and correctly, and faulty means that an abnormal state such as failure/fault occurs in the device.

The pipe diameter is the diameter of the pipe where the water meter is installed, and it has a value between 15–100 mm depending on whether it is used in a single household or a commercial building. The AMI battery voltage stores the voltage value of the AMI battery, and a voltage value between 3.4–3.6 V or 0 V is stored. The cumulative water flow is the total accumulated water flow measured from the time when the water meter is first installed to the current measurement time, and it corresponds to the cumulative water consumption of the customer. The current water flow is the water flow between the previous measurement time and the current measurement time, and it corresponds to the amount of water used by the customer during the corresponding time interval. The unit of cumulative water flow and current flow is tonnes.

### 2.3. AMI Data Challenges

The collected/stored AMI data is real-world data. There are many challenges in applying these data directly to train the ML model, which are listed as follows:

- **Changes in the number of customers over time:** We do not have AMI data for the entire period (360 days) for all customers because the RWMR devices were gradually installed over time.
- **Unclear location of failure/fault in the RWMR device:** The RWMR device is a combination of the remote water meter and the AMI remote meter reader, and both devices can fail. However, the collected AMI data does not indicate which part of the two devices has failed.
- **A large amount of missing data:** Empty data is frequently stored when the RWMR device fails or a (wireless) communication problem occurs.
- **Inconsistency in the time interval:** Each customer’s water flow (consumption) should be measured every hour on the hour, but the measurement is often not recorded every hour. There are cases where the measurement time is shorter than an hour (e.g., 50 min) or longer than an hour (e.g., 70 min).
- **Many cases where the current flow value is zero:** There are instances wherein current water flow may be incorrectly recorded as zero owing to a failure/fault of the remote water meter reading device.
- **An insufficient number of meaningful features:** Our AMI data does not have a sufficient number of meaningful features because of battery consumption, efficient communication, and other hardware issues.
- **A large imbalance between healthy and faulty data:** The number of healthy data is much higher than the number of faulty data. Therefore, it is difficult to train the ML model.

## 3. Data Preprocessing

The AMI data used in this study is a real-world dataset, and because of the challenges mentioned above, it cannot be used directly to train the ML model and must undergo extensive preprocessing steps. The following three preprocessing steps are sequentially performed in order: data cleaning, data alignment, and missing value estimation.

### 3.1. Data Cleaning

The cumulative water flow is a function that increases with time because it corresponds to the cumulative water consumption of the corresponding customer. Therefore, the cumulative water flow at the current measurement time cannot be less than the cumulative water flow at the previous measurement time. However, such paradoxical cases do occur in actual AMI data. First, if the cumulative water flow at the current measurement time is smaller than the cumulative water flow of the previous measurement time, the current water flow is corrected to zero, and the cumulative water flow at the current measurement time is corrected to be the same as the cumulative water flow of the previous measurement time.

Second, if the cumulative or current water flow is not recorded for a long period, possibly owing to a failure/fault of the device or a communication problem, those data are removed. Specifically, if the cumulative or current water flow is not recorded more than six times (i.e., 6 h or more), the corresponding data is removed.

Finally, there are cases where AMI data is normally stored every hour on the hour, but one or more additional AMI data are stored in between. In this case, only the AMI data that came in on time are retained; the additionally recorded AMI data are considered to be unnecessary and removed. Furthermore, when those AMI data are removed in this manner, the removed current water flow is added to the current water flow of the next measurement time data. Table 1 presents one such example. In this example, there exist additionally received AMI data at 12:10, not on the hour (i.e., 12:00), and it is removed and the current water flow at 13:00 is corrected.

### 3.2. Data Alignment

Our AMI data is collected/stored at the server every hour on the hour. However, if the measurement time interval is not one hour, it should be corrected. In this case, the cumulative water flow is first corrected by using the most basic and simple interpolation method, a linear interpolation method. After correcting the cumulative water flow, the current water flow is also corrected using the following equation:

(1) $$\mathrm{Updated}\;\mathrm{current}\;\mathrm{water}\;\mathrm{flow}=\frac{\mathrm{Current}\;\mathrm{water}\;\mathrm{flow}}{\mathrm{Time}\;\mathrm{interval}}.$$

The cumulative water flow of the current measurement time affects the current water flow of the next measurement time. Finally, using the corrected cumulative water flow of the current measurement time, the current water flow of the next measurement time is also corrected.

Table 2 presents an example. In this example, there is no AMI data collected at 13:00, but there is AMI data collected at 13:15, so the cumulative water flow at 13:00 is first computed using linear interpolation, and then the current water flow at 13:00 is also computed using (1). Finally, the current water flow at 14:00 (next measurement time) is corrected using the difference between the cumulative water flow at 14:00 and the cumulative water flow at 13:00.

### 3.3. Missing Value Estimation

Our AMI dataset has a considerable amount of missing data. More than 50,000 AMI data (approximately 1.8% of the total AMI data) are missing. We observe that most of the missing values occur in the cumulative water flow, current water flow, and AMI battery voltage values. These missing values contain important information and can be caused by various reasons, such as failure/fault of the RWMR device, communication problems, and battery problems. In the case of data analysis using real data, estimation of missing values is very important. This is because if the missing values are not accurately predicted, the ML model can be trained using incorrectly predicted data, which causes the performance of the ML model to deteriorate.

The following method is proposed to accurately predict these missing values. First, the distribution of missing data in the actual AMI data is obtained. In the previous data-cleaning step, periods of missing data spread over six consecutive hours or more are removed, so there only exist periods of missing data spread over 1–5 h in a row. In our AMI dataset, the percentage of the missing data periods is 4.6% for 1 h, 80.8% for 2 h, 0% for 3 h, 12.8% for 4 h, and 1.8% for 5 h in a row. Second, in order to evaluate various missing value estimation methods, certain lengths of AMI data that are already populated with values are removed. The length of data to be removed is sampled based on the ratio of missing data obtained as described above. This sampling process is repeated 10,000 times to intentionally remove some AMI data with values. Third, various estimation methods are used to estimate the values of data that were intentionally removed in our previous step. Finally, the estimation method with the minimum error between the actual value and the predicted value is selected. We use a mean square error (MSE) metric for comparison. Here, MSE refers to the mean square distance between the actual and predicted values. The estimation method selected in the final step is used to predict the actual missing values in the AMI data.

**Figure 3:**
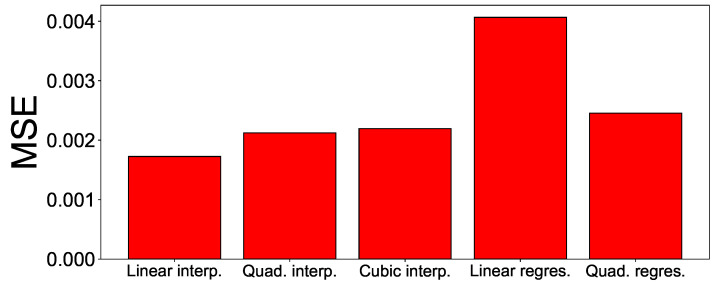
Mean square error results using various estimation methods for missing value estimation. Five estimation methods are compared.

Five estimation methods are compared in the experiment. These are linear interpolation, quadratic interpolation, cubic interpolation, linear regression, and quadratic regression. The experimental results are shown in Figure 3. The experimental results show that the MSE value of linear interpolation is 0.001756, which showed the smallest MSE value compared to other methods. The MSE value of linear interpolation is approximately 23% smaller than the second-best method (i.e., quadratic interpolation method).

Table 3 presents an example of predicting missing values in our AMI data using the linear interpolation method. In this example, the collected AMI data includes the data at 11:00 and 14:00, but the data in between (i.e., 12:00 and 13:00) are missing. First, the cumulative water flows at the missing time points (i.e., 12:00 and 13:00) are interpolated and added using linear interpolation, and then the current water flow in the missing section is also added using the interpolated cumulative water flow at 12:00 and 13:00. Finally, using the cumulative water flow at 13:00 and 14:00, the current water flow at 14:00 is also corrected using the difference between the cumulative water flow at 14:00 and the cumulative water flow at 13:00.

## 4. ML Problem Formulation and ML Models

### 4.1. Problem Formulation

The AMI data is assigned one of two status codes: healthy (label 0) or faulty (label 1). Here, faulty means a state such as failure/fault of the RWMR device. It should be recalled that the RWMR device is a combination of the water meter for remote meter reading and the AMI remote meter reader, which can fail in both places. We formulate the problem of predicting faulty RWMR devices as a binary classification problem. Our goal is to accurately predict the healthy/faulty state of the RWMR device using ML models. We also propose a method to evaluate the performance of the ML model by considering the cost incurred owing to mispredictions made by the ML model.

**Figure 4:**
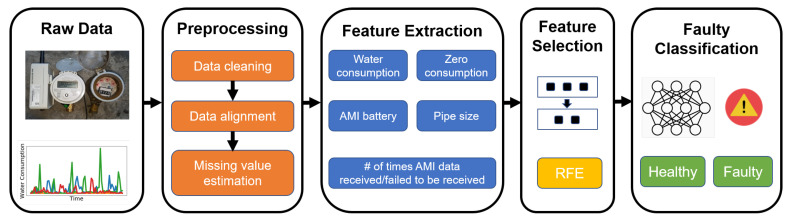
Proposed solution for predicting faulty RWMR devices.

The proposed solution for predicting faulty RWMR devices is summarized in Figure 4. It is composed of four steps. First, the extensive preprocessing methods described in Section 3 are performed on the raw AMI data. Second, feature extraction is performed to extract meaningful features. Data after feature extraction often becomes high-dimensional data. Third, feature selection is performed to select meaningful features that significantly affect the output class. Specifically, feature selection using recursive feature elimination (RFE) is performed. Finally, fault classification is performed using the ML model, and the AMI data is predicted as being either healthy (label 0) or faulty (label 1).

### 4.2. Feature Extraction

When predicting faulty RWMR devices using the ML model, it is good to have as many meaningful features as possible for classification. However, only limited information is stored in the raw AMI data. Our preliminary experiments show that the performance of the ML models greatly decreased when feature extraction is not performed. To this end, 23 meaningful features are extracted. The RWMR device is a combination of the water meter for remote meter reading and the AMI remote meter reader, and failures may occur in either place. Therefore, we extract meaningful features from both places.

In relation to the water meter, features related to water consumption and features related to the history of zero consumption of customers are extracted. Here, zero consumption indicates that the current flow is zero. Zero consumption is an important feature in predicting faulty meters because, in many cases, even if the customer actually uses water, zero consumption can be recorded for a long time because of the failure/fault of the device. Zero consumption may also be recorded in genuine cases, e.g., because the customer is away from home for a long time.

Regarding the failure/fault of the AMI remote meter reader, features related to the number of times AMI data were received or failed to be received for a specific period are extracted. Basically, AMI data is sent to the server every hour, but given that AMI data can be received repeatedly in the case of device malfunction, this feature is used for feature extraction. Finally, the AMI battery voltage value may suddenly drop when the battery of the AMI remote meter reader is turned off owing to the failure/fault of the AMI remote meter reader. The actual AMI battery voltage value and whether the voltage value of the AMI battery is zero are extracted. The extracted features are divided into five categories, which can be summarized as follows:

- Nine features related to the water consumption of the customer (e.g., current water consumption (flow) and standard deviation of water consumption over the last 24 h)
- Three features related to zero consumption (e.g., number of instances of zero consumption during a week, number of instances of zero consumption during a month, consecutive number of zero consumption events)
- Two features related to the AMI battery voltage value (e.g., AMI battery voltage value, binary number indicating whether the AMI batter voltage value is zero or not)
- One feature related to pipe size where the water meter is connected
- Eight features related to the number of receiving or failing to receive AMI data for a specific period (e.g., number of AMI data received over the last 24 h, number of times AMI data failed to be received over the last 24 h)

**Figure 5:**
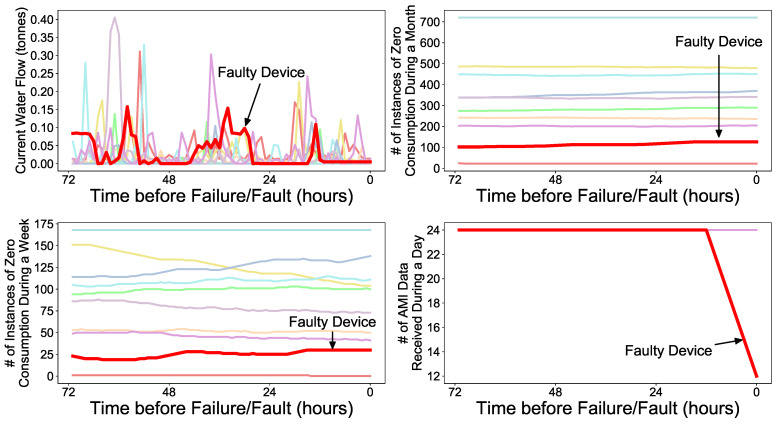
Extracted features of faulty (bold red) and healthy (other colors) devices until actual failure/fault of the RWMR device occurs.

Figure 5 shows an example of several extracted feature values until actual failure/fault of the RWMR device occurs. Here, bold red denotes the faulty device and the other colors are healthy devices. We observe that several extracted features of the faulty device show distinguishable patterns from healthy devices.

### 4.3. Feature Selection

After performing the feature extraction step, 23 features are extracted and it becomes high-dimensional data. We select features that have a higher influence on the label of the classifier (target) for improving the performance and efficiency of the ML model. The feature selection method used in this study is a recursive feature elimination (RFE). The RFE has been widely used successfully in applications such as genetics [7] and HDD failure detection [3].

The RFE learns a classifier (e.g., Support Vector Machine) that can weight a feature for a given feature set data, removes the feature with the lowest weight, and repeats this process to reach the desired number of features. However, the user has to determine the desired number of features. As an improved method, the number of features with the best classifier performance is obtained using cross-validation. We use the RFE to rank features and select the optimal number of features using cross-validation.

### 4.4. ML Models

Four ML models are used to predict the failure/fault of the RWMR device. The input of the ML model is *n* features, $x_{1}$, ⋯, $x_{n}$ and the output is a class *y* (0: healthy, 1: faulty). First, we use a random forest (RF) model, which shows good performance as a traditional ML model. In addition, a GMM-based model that shows good performance in HDD failure detection is used [3]. Our AMI data is a time-series data. We use a deep learning-based long short-term memory (LSTM) network model, which shows good performance in time-series data. Finally, a convolutional neural network with long short-term memory (CNN-LSTM), which has recently been proven to have strengths in feature extraction and prediction of time-series data, is used. We describe each ML model as follows:

- **RF [8]:** Random Forest (RF) is an ensemble learning-based classifier. It trains various decision trees using several training sets made by applying Bootstrap sampling to the training data, and passes new data through each tree simultaneously. Then it selects the final classification result of the data by voting on the classification result output by each tree. In this study, class *y* is predicted using the mean value (soft voting) of the predicted probability value of the tree.
- **GMM [3]:** GMM is a model that assumes that the data distribution is the sum of several Gaussian distributions. GMM models the probability for data *x* given by:
  (2) $$P\left(x|\theta \right)=\sum_{i=1}^{m}\pi_{i}N\left(x|\mu_{i},C_{i}\right),$$
  where ***x*** is a *n*-dimensional vector, and *m* is the number of Gaussian distributions. Here, $\pi_{i}$ is the weight of the *i*th Gaussian and $\sum_{i=1}^{m}\pi_{i}=1$. $N\left(x|\mu_{i},C_{i}\right)$ is a Gaussian probability density function with a mean vector $\mu_{i}$ and a covariance matrix $C_{i}$ and is defined as:
  (3) $$N\left(x|\mu_{i},C_{i}\right)=\frac{1}{\sqrt{{\left(2\pi \right)}^{n}\left|C_{i}\right|}}\exp\left(-\frac{1}{2}{\left(x-\mu_{i}\right)}^{T}C_{i}^{-1}\left(x-\mu_{i}\right)\right).$$
  The model parameter θ is the set of all parameters of *m* Gaussian distributions, $\left\{\pi_{i},\mu_{i},C_{i}\right\}$ where *i* = 1, ⋯, *m*. The GMM model used in this study is basically similar to the GMM model used for HDD failure detection in a previous study [3]. The GMM-based baseline model building process and failure/fault prediction process involves the following three steps.

The first step is to fit the GMM through the EM algorithm using only some of the healthy data. The EM algorithm is a technique for estimating the parameters of the probability distributions when the distribution of the dataset represents the parametric probability distributions. The user must decide how many Gaussian distributions should be expressed for healthy data. In this study, the number of Gaussian distributions to use is determined using the Bayesian Information Criterion (BIC) score as shown below. The BIC score is one of the criteria to balance the log-likelihood function and model complexity, and is defined as follows:

(4) $$\mathrm{BIC}=-2\cdot \ln\left(\hat{L}\right)+r\cdot \ln\left(M\right),$$

where $\hat{L}$ is the likelihood of the dataset for the probabilistic model, *r* is the number of parameters in the model, and *M* is the size of the dataset. A lower BIC score indicates a better model.

The second step is to determine the likelihood baseline that divides the healthy and the faulty data. For the GMM, likelihood values of healthy and faulty data of the training data are obtained, and the likelihood baseline is determined based on them. This baseline is determined by the user in consideration of the trade-off between false alarm rate (FAR) and failure detection rate (FDR). Finally, when new data comes in at the last step, the likelihood of the GMM distribution obtained in the first step is calculated, and class *y* is predicted by checking whether it is less than the likelihood baseline obtained in the second step.

- **LSTM [9,10]:** LSTM is a deep learning model in which the cell state is added to the hidden state of the recurrent neural network (RNN). It is proposed in order to solve the vanishing gradient problem that occurs when the length of the input sequence in the RNN increases. The cell state in the LSTM is like a memory, and even if the state has existed for a long time, it preserves the information for a long time and allows the gradient to propagate well. LSTM is known to achieve good performance in processing time-series data.

Disk failure prediction using LSTM has been studied earlier [9]. Unlike the above models in which the extracted features of 1 h are inserted, the input of several hours is bundled using windowing and given as the time-series input of the LSTM to exploit the superiority of the time-series processing capability of LSTM.

- **CNN-LSTM [11]:** CNN-LSTM is a method of spatio-temporal deep learning that utilizes the temporal features of sequences as well as CNNs to extract features. It is a model that combines a CNN, which has strength in extracting features between adjacent space and time, and LSTM, which has strength in processing time-series data. CNN-LSTM is divided into four layers, i.e., an input layer, a convolutional network layer, an LSTM layer, and an output layer. A recent study used a CNN-LSTM structure for HDD failure prediction [4]. However, our study is the first to use a CNN-LSTM structure to predict the failure/faults of RWMR devices from real-world AMI data.

## 5. Datasets and Metrics

### 5.1. Datasets

The AMI dataset used in this study was collected by the IS technology system. The collection period of AMI data was from 15:00 on 1 April 2020, to 12:00 on 26 March 2021, i.e., 360 days. RWMR devices were installed for 2762 customers in some areas of small-sized cities in Korea, and AMI data were collected/stored. However, AMI data is not available for all customers for all periods of 360 days because the number of customers who installed the devices gradually increases instead of all devices for all customers being installed at once. In our study, the experiment is conducted on customers who had collected AMI data for at least 6 months. The collected AMI data stores the water meter diameter, which is related to usage. The 15-mm diameter is for household use only, while the 20-mm or larger diameters can be used for household and commercial use. Approximately 85% of customers use a 15-mm pipe diameter and the rest use a 20-mm or larger pipe diameter.

After performing extensive preprocessing steps described in Section 3, 2,284,980 healthy data and 978 faulty data are used in the experiment. However, the ratio of healthy and faulty data is highly unbalanced and the amount of faulty data is too small compared to the healthy data. It is difficult for the ML models to learn these highly unbalanced data. Especially for the GMM model, the EM algorithm needs several parameters and the time of convergence of these parameters also becomes too long when the number of data is too large.

To solve these problems, we choose healthy and faulty data in different ratios when training the ML model. Specifically, only 20% of healthy data and 50% of faulty data are used for training, and all remaining data are used for testing. When the ratio of healthy data for training is very low (e.g., 5%), we observe that the ML models fail to learn the healthy data properly, so although the recall slightly increases, the precision decreases significantly. By contrast, when the ratio of healthy data is very high (e.g., 50%), there is no significant difference in performance, but only the training time increases. When the ratio of faulty data is set as high as 80%, we observe that the results fluctuate depending on which data is selected for the test because there are too few faulty data during the test. By contrast, when the ratio of faulty data is very low (e.g., 20%), we observe that the performance significantly decreases because the ML models fail to learn the faulty data properly. Furthermore, the training and test data are chosen by random sampling without considering the customer ID. Similar to a previous study [12], only faulty data are oversampled by a factor of 200 to the training data.

### 5.2. Hyperparameters

**Figure 6:**
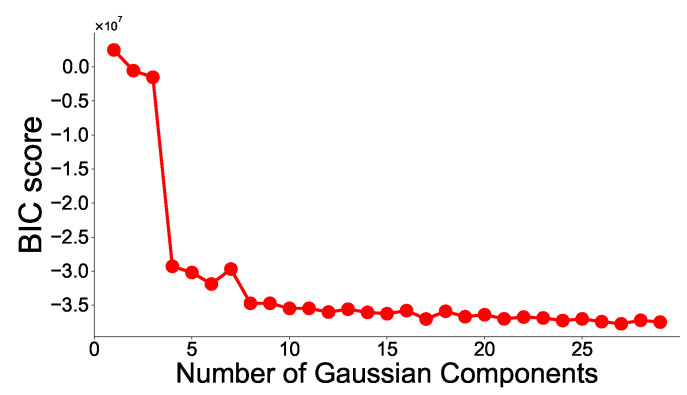
BIC Score with respect to the number of Gaussians components on the training dataset.

In the case of the RF model, the number of trees in the forest is set to 100, and the max depth of the tree is set to four. In the case of the GMM model, the number of Gaussians to fit the baseline is determined by the BIC score. The experimental result is shown in Figure 6. We observe that the BIC score is minimum when the number of Gaussians is 27. Therefore, the number of Gaussians is selected as 27.

For LSTM models, the previous 72 h at the prediction time are used as the time-series input of the LSTM. Here, one step corresponds to 24 h. The first step is from the previous 71 h to the previous 48 h, the second step is from the previous 59 h to the previous 36 h, and so on. Finally, the fifth step is from the previous 23 h to the prediction time. At every step, 16 feature data (i.e., number of features) of 24 h are flattened as input, and the input with (5, 384) dimension comes in one inference. Furthermore, for the unit, the best parameter is found by performing a grid search. As a result, we create an LSTM model having a first-layer LSTM layer with 128 nodes and a first-layer multi-layer perceptron (MLP) layer that outputs one failure probability. The LSTM layer uses a tanh activation function and the MLP layer uses a sigmoid activation function. An Adam optimizer is used as in MLP and regularized using an L2 penalty of 0.01. The learning rate is given as a constant of 0.001. An epoch of 10 is used because the loss converges after 10 epochs.

**Figure 7:**
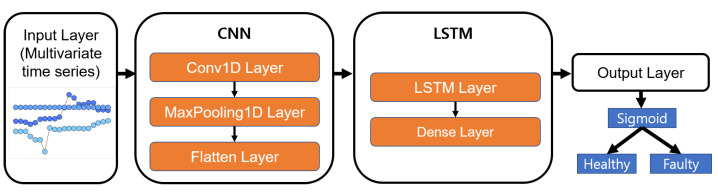
Proposed CNN-LSTM model.

In the CNN-LSTM model, similar to the LSTM model, time-series input of several times is given using windowing. The CNN-LSTM model receives time-series input in the same way as the LSTM model. There are various modified versions in addition to the basic form of CNN-LSTM, such as regional CNN-LSTM, which handles sentences of various lengths, CNN-BiLSTM, which considers bidirectional context, and CNN-GLU, which replaces LSTM with GLU. In our study, we have used a basic CNN-LSTM model that combines 1D CNN and unidirectional LSTM. The CNN-LSTM model used in our study is shown in Figure 7. First, a CNN with the same parameters is used for every step, and the time-channel dimension is flattened to one dimension and input to the LSTM. Our CNN-LSTM consists of a 1D convolution layer, a Max-Pooling layer, a flatten layer, an LSTM layer in the first layer, and finally an MLP layer in the first layer that outputs the failure probability. The 1D convolution layer has 16 filters with a size of four, and uses valid padding and ReLU activation. The Max-Pooling layer has a size of two and a stride of two. LSTM uses 256 nodes and tanh activations. The MLP layer outputs the failure probability using the sigmoid activation function. An Adam optimizer is also used, and all layer parameters are regularized with an L2 penalty of 0.001 L2 parameters. The learning rate is given as a constant of 0.001. Similar to the LSTM model, an epoch of 10 is used.

### 5.3. Performance Metrics

To evaluate the performance of the ML models, we use several performance metrics: precision, recall, F-measure, Matthews correlation coefficient (MCC), and receiver operating characteristic (ROC) curve. We briefly describe these metrics as follows.

- **Precision [13]:** Precision is defined as the percentage of the faulty data among those predicted to be faulty (positive). It is calculated by the following formula:
  (5) $$\mathrm{Precision}=\frac{\mathrm{TP}}{\mathrm{TP}+\mathrm{FP}},$$
  where TP is the number of actually faulty data that are correctly predicted as faulty and FP is the number of actually healthy (negative) data that are incorrectly predicted as faulty.
- **Recall [13]:** Recall is the percentage of the faulty data correctly identified among the actually faulty data. It is calculated using the following formula:
  (6) $$\mathrm{Recall}=\frac{\mathrm{TP}}{\mathrm{TP}+\mathrm{FN}},$$
  where FN is the number of actually faulty data that are incorrectly predicted as healthy.
- **F-measure [14]:** Our dataset is a highly unbalanced dataset where the number of healthy data is much higher than the number of faulty data. In such a scenario, F-measure, which is the harmonic mean of precision and recall, is often used. F-measure has a value between 0 and 1, where a higher value indicates better prediction. It is defined as:
  (7) $$F\text{-}\mathrm{measure}=\frac{2\cdot \mathrm{Precision}\cdot \mathrm{Recall}}{\mathrm{Precision}+\mathrm{Recall}}.$$
- **MCC [15]:** MCC is known as a metric more suitable for imbalanced data than F-measure [4]. MCC has a value between −1 and 1. When it is 1, it indicates perfect prediction, while when it is −1, it indicates inverse prediction. The MCC is defined as:
  (8) $$\mathrm{MCC}=\frac{\mathrm{TP}\cdot \mathrm{TN}-\mathrm{FP}\cdot \mathrm{FN}}{\sqrt{\left(\mathrm{TP}+\mathrm{FN}\right)\left(\mathrm{TP}+\mathrm{FP}\right)\left(\mathrm{TN}+\mathrm{FP}\right)\left(\mathrm{TN}+\mathrm{FN}\right)}},$$
  where TN is the number of healthy data that are correctly predicted as healthy.
- **ROC curve:** The ROC curve is the most commonly used performance measurement method for binary classification problems and shows a trade-off between the FAR and the FDR [16]. FDR refers to the ratio of detected faulty data among all faulty data and corresponds to the true positive rate (TPR). It is computed as:
  (9) $$\mathrm{FDR}=\frac{N_{FD}}{N_{F}},$$
  where $N_{F}$ is the total number of faulty data and $N_{FD}$ is the number of detected faulty data. FAR is the percentage of data for which a false alarm occurred among total healthy data and corresponds to the false positive rate (FPR). It is computed as:
  (10) $$\mathrm{FAR}=\frac{N_{FA}}{N_{H}},$$
  where $N_{H}$ is the total number of healthy data and $N_{FA}$ is the number of data for which a false alarm occurred. The ROC curve is a two-dimensional graph, with FAR on the *x*-axis and FDR on the *y*-axis. FAR and FDR depend on what thresholds are set, and this is indicated on the ROC curve.

### 5.4. Threshold Selection

In most cases of HDD failure detection, FDR performance is evaluated at 0% FAR or low FAR. This is because HDD manufacturers prefer to reduce FAR because of warranty issues [3,17,18]. In this paper, we propose a method to evaluate the performance of ML models using two threshold selection methods.

First, similar to the previous HDD failure detection problem, the performance of ML models at low FAR is evaluated. Specifically, the threshold is set to FAR = 0.1%. In the case of the dataset used for the experiment, the total number of our AMI data is approximately 2,280,000, and the FAR = 0.1% standard gives approximately 2280 total false alarms (FP) per year. As the number of customers used in the experiment is 2762, false alarms occur approximately once a year per customer. This number of false alarms is considered to be an acceptable number for water suppliers and customers.

Second, we propose a method to set a threshold for evaluating the performance of ML models in terms of total cost. In case of a false alarm, the cost of the (device) manufacturer increases when a technician is unnecessarily sent. By contrast, false negatives (FN) may cause NRW because the ML model fails to predict the actual failure/faults of the device. If the model fails to predict the actual failure/faults, less water consumption may be recorded in the water meter than the amount used by the customer, and zero consumption may be recorded even though water is actually used. FPs and FNs are not good for manufacturers and water suppliers. From the manufacturer’s point of view (e.g., IS technology), we consider FNs to be a more serious problem than FP because, in the case of false alarms, the customer can tolerate a certain number of false alarms and does not complain about an acceptable number of false alarms even if the predicted results are not correct. Furthermore, in such a case, the manufacturer can send a technician only if multiple alarms occur in a row.

Thus, there is a trade-off between FP and FN in terms of cost [4]. Let $P_{1}$ and $P_{2}$ be the ratios of healthy and faulty data among all data, receptively. The cost *C* can be defined as follows:

(11) $$C=w_{\mathrm{FNR}}\cdot \mathrm{FNR}\cdot P_{1}+w_{\mathrm{FPR}}\cdot \mathrm{FPR}\cdot P_{2},$$

where $w_{\mathrm{FNR}}$ and $w_{\mathrm{FPR}}$ are defined as weights corresponding to FNR and FPR, respectively. Here, false negative rate (FNR) and false positive rate (FPR) is defined as $\mathrm{FNR}=\mathrm{FN}/\left(\mathrm{FN}+\mathrm{TP}\right)$ and $\mathrm{FPR}=\mathrm{FP}/\left(\mathrm{FP}+\mathrm{TN}\right)$, respectively. A smaller value of *C* indicates lower cost. The performance of ML models is evaluated by setting the threshold of FAR that minimizes (11). The weight can be adjusted according to the policy of the manufacturer or water supplier. As mentioned earlier, we set $w_{\mathrm{FNR}}$ to be much larger than $w_{\mathrm{FPR}}$ because FNR is much more serious than FPR for the manufacturer. As the *C* value can have a different value range for each ML model, the *C* value in (11) is normalized between 0 and 1.

## 6. Results

### 6.1. Feature Selection

**Figure 8:**
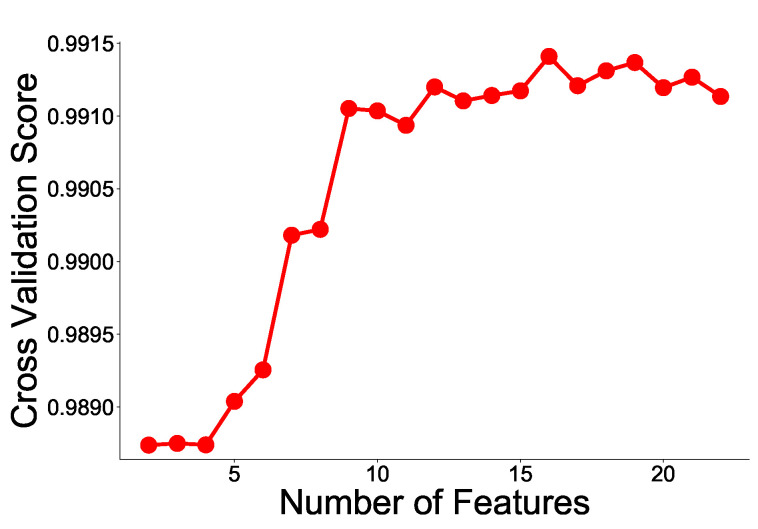
RFE cross-validation score with respect to the number of features.

Feature selection is performed on the AMI data using the RFE method described earlier. When using the RFE method, the random forest (RF) method is used, and each RF is trained using a two-fold cross-validation with 5, 10, 20, 25, and 50 trees. The experimental results show that there is no significant difference in the RFE results by when changing the number of trees is changed. Therefore, we use an RF model with 50 trees. The results of two-fold cross-validation of RFE are shown in Figure 8. As shown in the figure, the cross-validation score is the highest when the number of features is 16. Therefore, we chose the number of features to be 16. The reason for the significant improvement in the ninth feature is that the ninth feature is a completely new feature, unlike the existing eight features. The cross-validation score increases significantly when new features different from existing features are added. In Table 4, the top eight features are all related to the AMI remote meter readers, but the ninth feature is a completely new feature that is related to water meters for remote meter reading.

Table 4 lists the top ten selected features and their importance. The higher value indicates more important features. Overall, the features related to the number of times AMI data is received or fails to be received are selected as important features. The most important feature selected is number of times AMI data failed to be received during a week. In other words, a large number of instances of AMI data failing to be received for a specific period (especially for a long period of time) indicates that there is a high probability that the RWMR device is failed/faulty. Overall, the features related to the AMI remote meter reader are selected as the important features compared to the features related to water meters for remote meter reading. Therefore, we expect that many RWMR device failures/faults occur on the AMI remote meter reader side in the dataset used. Among the features related to the water meter, the current water flow and the number of instances of zero consumption per month are selected as important features.

### 6.2. Prediction Results

#### 6.2.1. Threshold with FAR = 0.1%

**Figure 9:**
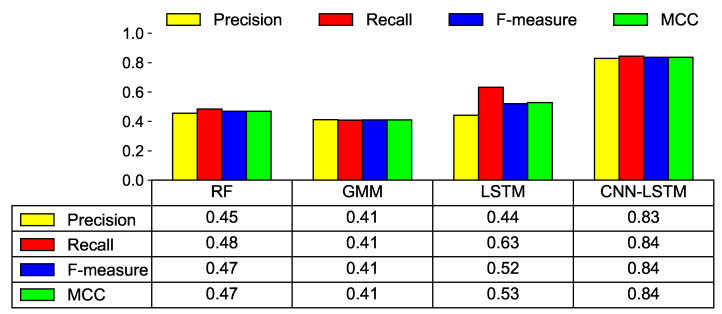
Model prediction results using various performance metrics when FAR = 0.1%.

Figure 9 shows the prediction results of various ML models when the threshold is set to FAR = 0.1%. The experimental results indicate that the CNN-LSTM shows the best performance among ML models. CNN-LSTM has an F-measure of 0.84 and an MCC of 0.84. The precision and recall of CNN-LSTM are 0.83 and 0.84, respectively. Among the compared ML models, the GMM shows the worst performance with an F-measure of 0.41 and an MCC of 0.41. Unlike other ML models, GMM performs baseline modeling using only healthy data and GMM cannot use the features of faulty data. Therefore, it demonstrates poor performance when faulty data has more prominent features than healthy data at a very low FAR compared to other models. We also observe that the performance degradation is significant in all ML models when only the first and second features of Table 4 were used for training. Specifically, when the CNN-LSTM model was used, the precision, recall, F-measure, and MCC were observed to drop significantly from 0.83 to 0.13, 0.84 to 0.54, 0.84 to 0.20, and 0.84 to 0.26, respectively.

**Figure 10:**
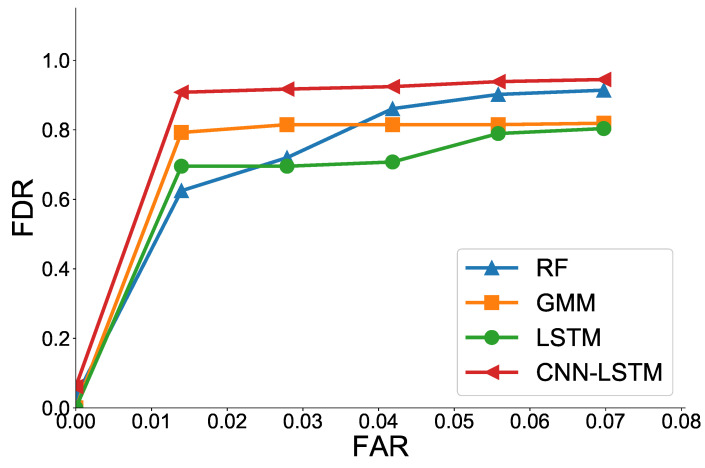
ROC curves of various ML models at low FAR.

Figure 10 shows the ROC curves of various ML models. For practical use, we focused on the performance, especially when the FAR is low (FAR ≤ 7%). The CNN-LSTM model also shows the best performance with the highest FDR for all FAR values. Specifically, when FAR = 7%, the CNN-LSTM achieves FDR values of 0.94, while RF achieves 0.91, MLP achieves 0.85, GMM achieves 0.82, and LSTM achieves 0.80. Interestingly, as the FAR value increases, we observe that the performance of the RF improves while the LSTM performance is relatively poor. The LSTM shows better performance than the RF at very low FAR, because time-series analysis of the LSTM shows strength in detecting some faulty data. The reason LSTM is not as good as other models at high FAR is the excessive generalization due to too much unnecessary information from the previous time in classifying healthy and faulty data. The CNN-LSTM model shows the best performance because it can select and provide only necessary time-series features through convolution.

#### 6.2.2. Cost-Effective Threshold

**Figure 11:**
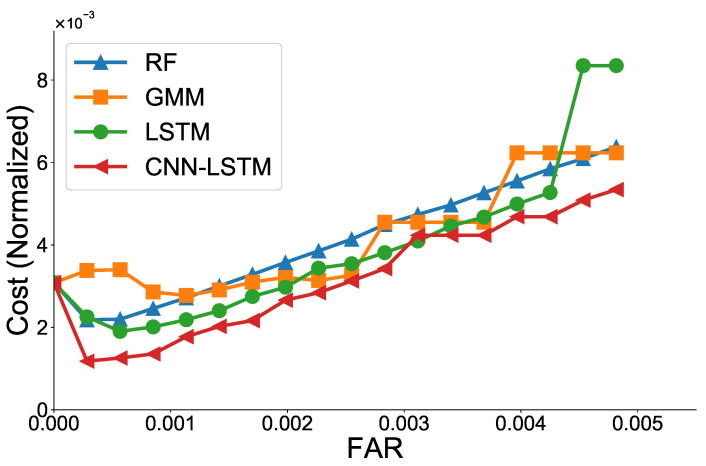
Normalized cost with respect to FAR for various ML models.

In this experiment, the weights $w_{\mathrm{FNR}}$ and $w_{\mathrm{FPR}}$ in (11) are set to 0.9 and 0.1, respectively, to assign a higher penalty to FNR than FPR. For practical use, the actual cost rather than the weight can be used, and a different ratio can be set depending on the policy of the manufacturer and the water provider. Figure 11 shows the normalized cost (*C*) of the ML models according to the FAR value. We observe that the FAR value with the minimum cost is different for each ML model. Overall, the CNN-LSTM model has the lowest cost in most cases. Table 5 presents the FAR value with the minimum cost for each ML model. The RF has a FAR value of 0.01% and GMM has a FAR value of 0.11% when the cost (*C*) is minimum.

**Figure 12:**
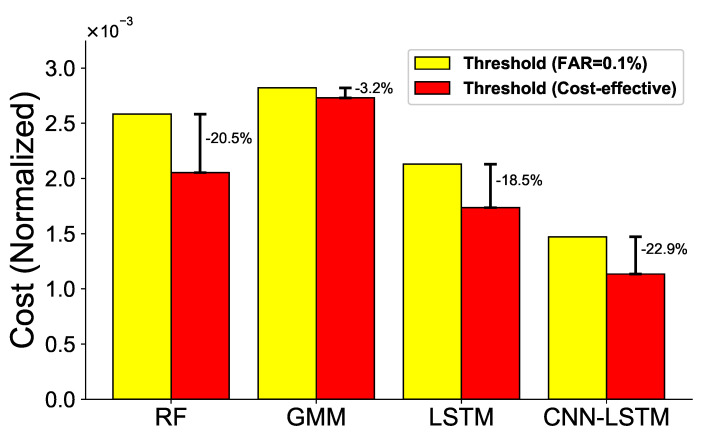
Comparison of normalized cost between fixed threshold (FAR = 0.1%) and cost-effective threshold.

Figure 12 shows the cost reduction associated with the proposed cost-effective threshold compared to the fixed threshold (FAR = 0.1%). We observe that the cost reduction ratio for each model is different. The cost reduction ratio of using the cost-effective threshold is 20.5% in the case of the RF and 22.9% in the case of the CNN-LSTM compared with the threshold of FAR = 0.1%.

**Figure 13:**
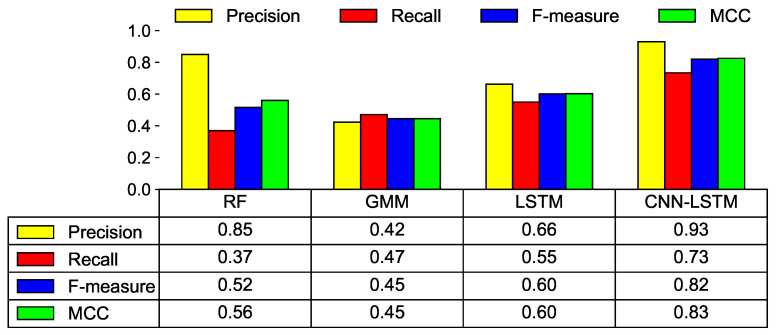
Model prediction results using various performance metrics with the minimum cost.

In Figure 13, for each ML model, the performance of the ML models is compared by setting the threshold as the FAR value when the minimum cost is obtained (see Table 5). The experiment demonstrates that the performance of the CNN-LSTM model is the best. F-measure shows a performance of 0.82 and an MCC of 0.83. Again, the GMM shows the poorest performance in most metrics. As mentioned earlier, the GMM builds a baseline using only healthy data; it does not learn the features of faulty data properly.

#### 6.2.3. Computation Time

We also compare the computation efficiency among various ML models to evaluate whether each ML model can be used practically. We measure training and inference time. The training and inference time of various ML models are presented in Table 6. All experiments are conducted in a Python3 environment using one AMD Ryzen 7 3800X and one NVIDIA GeForce RTX 2080 SUPER. For RF and GMM models, the scikit-learn package is used without using GPU [19], because the scikit-learn package does not support GPU. For LSTM and CNN-LSTM models, the TensorFlow 2 package is used with GPU [20].

We observe that the training time and inference time of deep learning-based ML models (LSTM and CNN-LSTM) take relatively longer than the traditional ML models (RF and GMM), as expected, but the actual inference time per sample is quite low for all ML models. The RF is the fastest in training and inference time, and the CNN-LSTM is the slowest. There is no significant difference in training time and inference time between the two deep learning-based models, LSTM and CNN-LSTM. Inference time per data sample of LSTM and CNN-LSTM is quite low in that it only takes approximately $5\times 10^{-5}$ seconds for inference. They take approximately 5 s to predict the label (class) of 100,000 data sample. In the case of CNN-LSTM, there is a trade-off between complexity and performance. When a more complex structure is used, the overall performance improves, but the complexity increases and so does the time taken.

## 7. Conclusions

We propose a CNN-LSTM-based solution that can predict faulty RWMR devices after collecting/storing actual water AMI data in South Korea. Our experimental results show that, first, our proposed preprocessing steps are effective in handling a challenging real-world dataset with limited features, and the extracted features are meaningful features that can predict the faulty devices. Second, we observe that the features related to the AMI remote meter reader are selected as the important features on the failure/fault of the device compared to the features related to water meters for remote meter reading. Specifically, features that have the maximum influence on the failure/fault of the device are the features related to the number of instances of receiving or failing to receive AMI data during a specific period. Third, we observe that the CNN-LSTM model shows the best performance among various ML models in predicting the failure/fault of the RWMR device. Specifically, it has an F-measure of 0.82 and MCC value of 0.83 when the cost-effective threshold is used. Finally, we also observe that the cost can be reduced by 22.9% when the cost-effective threshold is used compared with the fixed threshold when the CNN-LSTM model is used.

The RWMR device is divided into two parts: a water meter for remote meter reading and an AMI remote meter reader. Currently, the AMI data does not record in which of the two parts the failure/fault occurs. In future work, we plan to collect/store AMI data on which parts of the two devices have the failures/faults and to predict which part actually has a problem. We also plan to study solutions for minimizing NRW by predicting indoor leaks or pipe leaks using ML models.

## Figures and Tables

**Table 1:** Example of data cleaning. The colored AMI data is removed and updated using the proposed data cleaning methods.

	Measurement	Status	Pipe	AMI Battery	Cumulative	Current
	Time		Size	Voltage	Water Flow	Water Flow
**Raw data**	12:00	0	15	3.6	150.11	0.02
	12:10	0	15	3.6	150.12	0.01
	13:00	0	15	3.6	150.13	0.01
**Updated data**	12:00	0	15	3.6	150.11	0.02
	13:00	0	15	3.6	150.13	0.02

**Table 2:** Example of data alignment. The colored AMI data is updated using the proposed data alignment methods.

	Measurement	Status	Pipe	AMI Battery	Cumulative	Current
	Time		Size	Voltage	Water Flow	Water Flow
**Raw data**	12:00	0	20	3.4	4.10	0.10
	13:15	0	20	3.4	4.65	0.55
	14:00	0	20	3.4	4.85	0.20
**Updated data**	12:00	0	20	3.4	4.10	0.10
	13:00	0	20	3.4	4.54	0.44
	14:00	0	20	3.4	4.85	0.31

**Table 3:** Example of missing value estimation. The colored AMI data is estimated using the proposed missing value estimation methods.

	Measurement	Status	Pipe	AMI Battery	Cumulative	Current
	Time		Size	Voltage	Water Flow	Water Flow
**Raw data**	11:00	0	20	3.4	11.50	0.10
	14:00	0	20	3.4	11.53	0.03
**Updated data**	11:00	0	20	3.4	11.50	0.10
	12:00	0	20	3.4	11.51	0.01
	13:00	0	20	3.4	11.52	0.01
	14:00	0	20	3.4	11.53	0.01

**Table 4:** Top ten selected features and their importance. The highlighted feature is the most important feature selected.

Features	Importance
# of times AMI data failed to be received during a week	0.195
# of AMI data received during a week	0.190
# of AMI data received during two weeks	0.141
# of times AMI data failed to be received during two weeks	0.137
# of times AMI data failed to be received during a month	0.100
# of AMI data received during a day	0.064
# of times AMI data failed to be received during a day	0.061
# of AMI data received during a month	0.035
Current water flow (consumption)	0.013
# of instances of zero consumption during a month	0.012

**Table 5:** FAR values with the minimum cost for various ML models.

	RF	GMM	LSTM	CNN-LSTM
FAR	0.01%	0.11%	0.03%	0.03%

**Table 6:** Comparison of training time and inference time (per data sample) using various ML models.

	RF	GMM	LSTM	CNN-LSTM
Training time	30.03 s	125.01 s	373.61 s	388.24 s
Inference time	$0.36\times 10^{-5}$ s	$0.56\times 10^{-5}$ s	$5.00\times 10^{-5}$ s	$5.31\times 10^{-5}$ s

## Data Availability

The datasets used for this study are partially available on request to the corresponding author.
